# Neutrophil-to-lymphocyte ratio (NLR) as a predictive index for liver and coagulation dysfunction in preeclampsia patients

**DOI:** 10.1186/s12884-022-05335-1

**Published:** 2023-01-04

**Authors:** Hui Xing Cui, Chen Chen, Young Mi Jung, Zhen Yi Guo, Chun Yu Dong, Seung Mi LEE, Yin Hua Zhang

**Affiliations:** 1 Yanbian University Hospital, Yanji, Jilin Province China; 2 Department of Physiology and Biomedical Sciences, Ischemic/Hypoxic Disease Institute, Seoul National University College of Medicine, Seoul, Korea; 3 Department of Obstetrics and Gynecology, Seoul National University Hospital, Seoul, Korea

**Keywords:** Pre-eclampsia (PE), Neutrophil–lymphocyte ratio (NLR), Liver function, Kidney function, Coagulation, Prediction value

## Abstract

**Background:**

Pre-eclampsia (PE) is a pregnancy disorder that is related to an enhanced immune response. Immune cell characteristics such as neutrophil or monocyte to lymphocyte ratios (NLR, MLR) are known to be related to kidney and liver dysfunction in hypertensive patients. Here, we aimed to analyze the correlations between NLR, MLR and platelet to lymphocyte ratio (PLR) and liver, renal and coagulation functional parameters and the impacts of these immune cell profiles to the prognostic significance in PE patients.

**Methods:**

Pre-delivery hematological and biochemical parameters of 320 first-time pregnant women registered at the Obstetrics Department of Yanbian University Hospital from 2016 to 2019 were analyzed retrospectively. Patients were divided into normal pregnancy (normal, *n* = 161), mild PE (mPE, *n* = 28) and severe PE (sPE, *n* = 131) groups according to diagnostic criteria. Pearson correlation analysis were performed and area under the curve (AUC) were conducted for the diagnostic values of NLR, MLR and PLR. Results were validated with data from the Department of Obstetrics and Gynecology of Seoul National University Hospital (SNUH).

**Results:**

Kidney functional indexes were adversative in mPE and sPE and liver and coagulation indexes were worse in sPE compared to normal groups. Among immune cells, lymphocytes were increased in mPE and sPE patients, resulted in reduced NLR, MLR and PLR in PE groups, more significant difference were shown in sPE. NLR and PLR were associated with CREA and/or BUN negatively and positive associations were observed with total protein (TP) and albumin (ALB) in sPE. Only NLR showed positive associations with coagulation indexes (PT and APTT) in sPE. AUC analysis for NLR, MLR and PLR were 0.700, 0.656, 0.643, respectively, and NLR < 3.7 predicted hypertension (95% CI in all participants: 0.647–0.749, *p* < 0.001). Blood pressure, liver, kidney and coagulation indexes were worse at cut off value (NLR < 3.7), and this was validated with the data from SNUH.

**Conclusion:**

NLR could be used as an independent predictor of liver and coagulation dysfunction in PE patients. Our results may provide non-invasive and efficient way of the risk assessment among PE patients.

## Background

Preeclampsia (PE) is one of the serious complications of pregnancy with an occurring rate of 3–3.5% from 20 weeks into pregnancy [1, 2]. The clinical manifestations of PE are hypertension, proteinuria, HELLP syndrome (hemolysis, elevated liver enzymes, and thrombocytopenia), maternal pulmonary edema, cerebral palsy, and cerebrovascular accidents in severe cases [3–6]. PE seriously threatens the life and health of mothers and babies; it is one of the main causes of maternal and perinatal illness and death. The etiology of PE has not been fully elucidated and there is no effective therapeutic strategy apart from trimester termination, anti-hypertensive therapy, and the use of MgSO_4_ to relieve spasms. It is well known that PE is closely related to maternal immune responses; characterized by a shift towards a pro-inflammatory state (increased pro-inflammatory immune cells and cytokines and reduced anti-inflammatory immune cells and cytokines [7, 8]).

Innate immune cells are shown to be increased in the circulation even during normal pregnancy, activation of which facilitates trophoblast invasion and uterine spiral artery remodeling [9–11]. In PE patients, pro-inflammatory cytokines (e.g. IL-6 and TNF-alpha) are increased [12, 13], excessive inflammatory stress causes oxidative stress, damage to vascular endothelial cells directly and vascular disorders, which are important precursors of hypertension and comorbidities of PE, including liver dysfunction and coagulation disorders [14].

Recently, the activities of neutrophils and lymphocytes are shown to play important roles in the development of PE [15]. As the inflammatory indicators, the neutrophil–lymphocyte ratio (NLR) and monocyte-lymphocyte ratio (MLR) are known to be sensitive markers of inflammatory response and to predict the prognosis of the disease [16–20]. In some studies, serum NLR from circulatory blood has been shown to be increased in the PE group compared to those in normal pregnancy [21–24]. But others have shown no difference in NLR between PE and normal pregnancy [25, 26]. On the other hand, platelet-to-lymphocyte ratio (PLR) are closely associated with liver and coagulation status [27]. Therefore, thorough understandings of the immune cell characteristics and the relationship of their changes with liver, kidney and coagulation functions are important in better understandings of the disease.

Here, we retrospectively analyzed the correlations between NLR, MLR, PLR and liver, kidney and coagulation functional indexes those were obtained from clinical examination results in PE patients and normal pregnant women. ROC curve analysis was performed to validate the prognostic value of NLR.

## Methods

### Study design and patient groups

We designed a retrospective study by analyzing a medical registry database of 320 first pregnancy women who were registered in the Department of Obstetrics, Yanbian University Hospital from August 2016 to August 2019. All the patients were from local community (Yanbian region, Jilin Province China). The patients were divided into the normal pregnancy group (normal, 161 patients), mild PE group (mPE, 28 patients), and severe PE group (sPE, 131 patients) according to the medical records. Database of normal pregnancy population were collected from normal delivery and cesarean section groups, whose examination results are complete during the period of 2016–2019 (which is consistent with those for mild and severe PE patients). Non-PE pregnancy population of cesarean section was patients with breech presentation, pelvic abnormalities, amniotic fluid abnormality (polyhydramnios or oligohydramnios) or cephalopelvic disproportion. Complications such as pregnancy diabetes, gestational heart disease, intrahepatic cholestasis, twins were excluded in the data collection.

### Preeclampsia diagnostic and exclusion criteria

Diagnoses of PE were based on reported American Congress of Obstetricians and Gynecologists (ACOG) clinical criteria. Specifically, mPE was diagnosed based on systolic blood pressure (SBP) ≥ 140 mm Hg and/or diastolic blood pressure (DBP) ≥ 90 mm Hg and/or proteinuria ( +) on two occasions at least 4 h apart and proteinuria (> 0.3 g per day) after 20-week gestation. sPE was diagnosed based on SBP ≥ 160 mm Hg or DBP ≥ 110 mm Hg or proteinuria > 5 g per day and at least one of the following clinical symptoms: pulmonary edema, microvascular disease, thrombocytopenia, impaired liver function, and peripheral severe organ involvement (visual impairment and headache). All patients were strictly classified according to the diagnostic criteria. These PE patients were excluded from the analysis: HELLP syndrome, essential hypertension, recent acute and chronic infections, premature rupture of membranes, and other internal surgical diseases.

### Collection and the analysis of clinical examination parameters

Peripheral blood samples were taken before the delivery. Specifically, the blood samples of sPE patients were taken 2–4 h before the delivery as most patients were admitted to the hospital soon after the diagnosis and the delivery was induced almost immediately. For mPE patients, the blood samples were taken on the next day after the hospital admission, therefore, the results were from 4–24 h before the delivery. For normal pregnancy (non-PE) patients, the blood samples were taken on the next day after the hospital admission and because the operation were scheduled the next day or the third day, the blood examination results were within 24–48 h before the delivery.

The clinical examination parameters from peripheral blood samples (collected upon admission) were measured using a Sysmex XN-1000Q automated blood analyzer. White blood cell (WBC), hemoglobin (HGB), platelet (PLT), neutrophil (Neu), lymphocyte (Lym), monocyte (Mon) counts were analyzed. NLR, MLR and PLR were calculated by dividing absolute Neu count, Mon count or PLT count by absolute Lym count. Roche basic automatic biochemical analyzer was used to detect kidney function (urea nitrogen, BUN; creatinine, CREA; carbon dioxide, CO_2_) and liver function (aspartate aminotransferase, AST; alanine aminotransferase, ALT; albumin, ALB; total protein, TP; total bilirubin, TBIL; direct bilirubin, DBIL). Coagulation function was examined with plasma prothrombin time (PT); Prothrombin time international normalized ratio (PTINR); thrombin time (TT); Activated partial thromboplastin time (APTT) and fibrinogen quantification (FBGC).

### Validation of the analysis results with external data source

Validation of the associations between inflammatory indexes and organ functional parameters were conducted by the Department of Obstetrics and Gynecology of Seoul National University Hospital (SNUH). We collected pregnant women with PE (*n* = 73) and normal pregnancy (*n* = 73) who were delivered in SNUH between 2016–2019 (similar period of those from Yanbian University Hospital). These women were divided into two groups (NLR <  = 3.7 and NLR > 3.7 groups) and the parameters (SBP, DBP, AST, ALT, TP, BUN, CREA, PT, APTT, FBGC) were compared. Ethical approval was obtained from the ethics committee of SNUH (IRB No. 2021–0542).

### Data analysis

Data analysis was performed using SPSS 26.0 statistical software. The blood pressure, liver, kidney and coagulation functional parameter data were expressed as Mean ± standard deviation. One-way ANOVA was used to compare between normal, mPE and sPE groups. Pearson correlation analysis was used to assess the correlations between NLR, MLR or PLR and liver, kidney and coagulation functional parameters. *P* < 0.05 was considered as statistically significant. ROC curve analysis further clarifies the predictive diagnostic value of NLR, PLR, and MLR.

### Justification for the study design

Inflammation affects cardiovascular systems and organ functions, and the severity and characteristics of the parameters implicate the PE progression. Immune cell counts and NLR, MLR, PLR are non-invasively available biomarkers of system inflammation, but the changes and their profile seem vary among studies. In addition, the correlations and significance of these inflammatory indexes in predicting organ functional status and the diagnosis of PE are not studied. Accordingly, we have designed to analyze systematically the inflammatory pattern in patients of normal, mPE and sPE, their correlations to impaired kidney or liver and coagulation functions. Furthermore, the impact of inflammatory indexes was validated in an independent medical sector to better define the significance of the study. Ethical approval was obtained from the ethics committee of both Yanbian University Hospital and Seoul National University Hospital. All the data were blindly checked by the students who were involved in the study.

## Results

### Clinical examination results in normal pregnancy, mPE and sPE patients

Table 1 showed the clinical examination results among three groups (P1: normal *vs.* mPE; P2: normal *vs.* sPE). Age was not different among three groups. But BMI was significantly higher in mPE and sPE compared to normal (BMI: P1 < 0.001, P2 < 0.001).

**Table 1:** Blood pressure and organ functional parameters of normal pregnancy, mPE and sPE patients

Variable		Normal	Mild PE	Severe PE	P 1	P 2
		**(*n* = 161)**	**(*n* = 28)**	**(n = 131)**		
**General info**	**Age (y)**	30.84 ± 4.33	30.32 ± 4.31	31.53 ± 5.57	0.605	0.224
	**BMI (kg/m**^**2**^**)**	28.12 ± 3.21	33.30 ± 4.32	31.28 ± 4.74	< 0.001*	< 0.001*
**BP**	**SBP (90–140 mmHg)**	113.96 ± 9.80	147.25 ± 9.06	160.27 ± 15.44	< 0.001*	< 0.001*
	**DBP (60–90 mmHg)**	71.49 ± 7.52	95.75 ± 8.31	102.53 ± 11.82	< 0.001*	< 0.001*
**Liver Function**	**AST (0–40 U/L)**	15.47 ± 7.17	15.25 ± 3.10	22.41 ± 16.45	0.927	< 0.001*
	**ALT (0–40 U/L)**	10.31 ± 8.05	9.86 ± 3.22	16.12 ± 19.93	0.876	< 0.001*
	**TP (60–83 g/l)**	63.36 ± 6.89	61.50 ± 3.82	58.79 ± 6.52	0.166	< 0.001*
	**ALB (37–53 g/l)**	36.99 ± 3.85	35.43 ± 2.78	32.67 ± 4.86	0.072	< 0.001*
	**TBIL (5.1–25.6 μmol/l)**	10.11 ± 4.27	7.54 ± 4.68	6.52 ± 5.29	0.009*	< 0.001*
	**DBIL (1.7–6.8 μmol/l)**	2.58 ± 1.42	2.10 ± 1.59	1.91 ± 2.02	0.164	< 0.001*
**Renal Function**	**BUN (2.5–7 μmol/l)**	2.79 ± 1.02	4.11 ± 4.02	3.86 ± 1.76	< 0.001*	< 0.001*
	**CREA (44–80 μmol/l)**	42.74 ± 7.35	50.61 ± 12.99	51.07 ± 15.84	0.002*	< 0.001*
	**CO**^**2**^** (21–29 μmol/l)**	21.70 ± 2.51	20.52 ± 2.64	19.14 ± 2.92	0.033*	< 0.001*
**Coagulation Function**	**PT (Sec)**	12.19 ± 0.72	12.25 ± 1.41	11.90 ± 0.78	0.701	0.003*
	**PT%**	117.90 ± 14.68	120.98 ± 25.15	126.22 ± 20.44	0.412	< 0.001*
	**PTINR**	0.92 ± 0.07	0.93 ± 0.14	0.90 ± 0.07	0.746	0.003*
	**APTT (Sec)**	31.31 ± 3.10	32.43 ± 4.43	32.38 ± 6.48	0.266	0.061
	**FBGC (g/l)**	4.87 ± 0.90	4.75 ± 0.72	4.87 ± 2.83	0.761	0.980
	**TT (Sec)**	15.10 ± 1.00	15.74 ± 1.53	15.67 ± 1.68	0.023*	< 0.001*

P1: normal *vs*. mPE; P2: normal *vs*. sPE

^*^P < 0.05 are statistically significant difference. Data expressed as mean ± standard deviation

As shown in Table 1, both systolic and diastolic blood pressures (SBP, DBP) were increased in mPE and sPE (P1 < 0.001, P2 < 0.001, respectively). In addition, kidney functional parameters (BUN and CREA) were significantly higher (BUN: P1 < 0.001 in mPE; P2 < 0.001 in sPE; CREA: P1 = 0.002 in mPE, P2 < 0.001 in sPE), and CO_2_ were lower (P1 = 0.033 in mPE and P2 < 0.001 in sPE) in PE patients.

Among liver functional parameters, AST and ALT were significantly higher in sPE (P2 < 0.001, P2 < 0.001, respectively, Table 1). TP and ALB were significantly lower in sPE (P2 < 0.001, P2 < 0.001, Table 1). TBIL was lower in both mPE and sPE (P1 = 0.009, P2 < 0.001) but DBIL was lower only in sPE (P2 < 0.001). Coagulation functional parameters (PT, PT% and PTINR) were significantly shorter in sPE (P2 = 0.003, P2 < 0.001 and P2 = 0.003, respectively, Table 1). However, TT was longer in mPE and sPE (P1 = 0.023, P2 < 0.001). These results indicate liver and coagulation changes in PE patients, more in sPE group.

### Hematology parameters in normal, mPE, and sPE patients

The WBC, HGB, PLT, Neu, Lym, and Mon counts were within normal ranges. Table 2 compared the hematological parameters of normal, mPE and sPE groups. Among these blood cells, only Lym count was significantly increased in mPE and sPE (P1 = 0.007, P2 < 0.001). As a result, Neu to Lym ratio (NLR), Mon to Lym ratio (MLR) and PLT to Lym ratio (PLR) were reduced in mPE patients (PLR: P1 = 0.06; NLR: P1 = 0.004; MLR: P1 = 0.06). Notably, PLR, NLR and MLR were significantly reduced in sPE (P2 < 0.001, respectively).

**Table 2:** Blood cell counts in normal pregnancy, mPE and sPE patients

Variable	Normal (*n* = 161)	Mild PE (*n* = 28)	Severe PE (*n* = 131)	P 1	P 2
WBC (4—10) × 10^9^/L	9.41 ± 2.38	9.43 ± 2.62	9.39 ± 2.39	0.958	0.943
RBC (3.5—5.5) × 10^9^/L	4.06 ± 0.40	4.15 ± 0.33	4.14 ± 0.46	0.274	0.073
HGB (110—160) g/L	119.06 ± 11.56	122.82 ± 11.85	121.91 ± 12.37	0.124	0.041*
PLT (100—300) × 10^9^/L	194.94 ± 54.97	201.75 ± 76.19	194.39 ± 64.19	0.586	0.937
NEU# (2—7.7) × 10^9^/L	7.12 ± 2.09	6.80 ± 2.04	6.71 ± 1.92	0.442	0.084
MON# (0.12—0.8) x 10^9^L	0.62 ± 0.18	0.64 ± 0.23	0.61 ± 0.22	0.592	0.856
LYM# (0.8—4) × 10^9^/L	1.63 ± 0.45	1.91 ± 0.58	1.97 ± 0.53	0.007*	< 0.001*
PLR	125.25 ± 42.81	109.78 ± 39.02	103.93 ± 40.10	0.069	< 0.001*
NLR	4.69 ± 2.29	3.66 ± 0.87	3.52 ± 0.95	0.004*	< 0.001*
MLR	0.41 ± 0.20	0.35 ± 0.12	0.32 ± 0.10	0.06	< 0.001*

P1: normal vs. mPE; P2: normal vs. sPE

^*^P < 0.05 are statistically significant difference. Data expressed as mean ± standard deviation

### Correlation between NLR, MLR and PLR with kidney, liver and coagulation functional parameters

As shown in the Table 3, NLR was negatively correlated with CREA (*r* = -0.222, *p* = 0.01) and positively correlated with TP (*r* = 0.172, *p* = 0.046) and ALB (*r* = 0.305, *p* < 0.001). NLR was positively associated with PT (*r* = 0.201, *p* = 0.019) and APTT (*r* = 0.202, *p* = 0.01). These results indicate that NLR could be a diagnostic index for liver and coagulation function in sPE patients.

**Table 3:** Correlation between PLR, NLR, MLR and liver, kidney and coagulation functions in sPE group

		**NLR**		**MLR**		**PLR**	
	**Index**	r	p	r	p	r	p
**sPE**	**AST(0–40 U/L)**	0.080	0.352	0.184	0.032*	-0.174	0.044*
	**TP(60–83 g/l)**	0.172	0.046*	0.044	0.609	0.213	0.013*
	**ALB(37–53 g/l)**	0.305	< 0.001*	0.196	0.023*	0.204	0.018*
	**BUN(2.5–7 μmol/l)**	-0.123	0.154	-0.030	0.727	-0.351	< 0.001*
	**CREA(44–80 μmol/l)**	-0.222	0.010*	-0.098	0.258	-0.264	0.002*
	**PT(Sec)**	0.201	0.019*	0.168	0.051	0.038	0.665
	**APTT(Sec)**	0.202	0.010*	-0.024	0.786	-0.174	0.043*

^*^P < 0.05 are statistically significant difference

MLR did not show correlations with kidney parameters, showed only weak but significant associations with AST (*r* = 0.184, *p* = 0.032) and ALB (*r* = 0.196, *p* = 0.023). However, similar to NLR, PLR was negatively correlated with BUN (*r* = -0.351, *p* < 0.001) and CREA (*r* = -0.264, *p* = 0.002) and positively correlated with TP (*r* = 0.213, *p* = 0.013) and ALB (*r* = 0.204, *P* = 0.018), indicating that PLR could predict kidney and liver functional changes in sPE. The association between PLR and APTT were negative in sPE (*r* = -0.174, *p* = 0.043).

### AUC of NLR, MLR, PLR

**Fig. 1:**
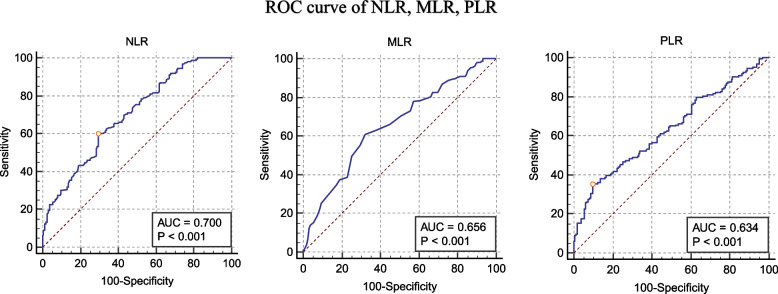
ROC curve of NLR, MLR, PLR

Area under the curve (AUC) analysis was performed to further clarify the diagnostic value of NLR, MLR, and PLR. As shown in the Table 4 and Fig. 1, AUCs of NLR, MLR and PLR were 0.700, 0.656 and 0.634 (95% CI = 0.647—0.749; 0.602—0.708 and 0.579—0.686, respectively). NLR showed higher specificity and sensitivity and the cut off value for NLR was < 3.7.

**Table 4:** Area under the curve (AUC) with optimal cut-off point for NLR, MLR, and PLR

	**NLR**	**MLR**	**PLR**
AUC	0.700	0.656	0.634
95% confidence interval	0.647–0.749	0.602–0.708	0.579–0.686
Cut off value	$\le$ 3.7	$\le$ 0.33	$\le$ 86.25
Sensitivity	59.51%	60.74%	34.97%
Specificity	70.37	67.90	90.12

### Evaluation of PE diagnostic value with NLR

Table 5 showed that according to NLR cut-off value (< 3.7 and > 3.7), the clinical examination results, *i.e.*, mean values of SBP, DBP, AST, ALT, TP, BUN, CREA indexes were significantly different, i.e. NLR < 3.7 group showed worse kidney, liver and blood pressure, which were diagnostic of PE.

**Table 5:** Difference of index between the NLR > 3.7 and NLR ≤ 3.7 (Yanbian University Hospital, CHINA)

Index	> 3.7 (*n* = 175)	$\le$ 3.7 (*n* = 145)	*P* value
SBP (90–140 mmHg)	130.9 ± 23.9	142.5 ± 26.1	< 0.0001(**)
DBP (60–90 mmHg)	82.4 ± 17.3	91.5 ± 17.2	< 0.0001(**)
AST (0–40 U/L)	16.8 ± 7.8	18.4 ± 8.6	0.0156(*)
ALT (0–40 U/L)	10.5 ± 5.1	12.7 ± 11.0	0.0175(*)
TP (60–83 g/l)	62.5 ± 5.2	60.3 ± 6.7	0.0010(*)
BUN (2.5–7 μmol/l)	3.0 ± 1.3	3.8 ± 2.3	0.0004(*)
CREA (44–80 μmol/l)	45.1 ± 10.6	49.3 ± 14.5	0.0031(*)
PT (Sec)	12.2 ± 0.9	11.9 ± 0.7	0.0034(*)
APTT (Sec)	31.7 ± 4.3	31.9 ± 5.6	0.7164
FBGC (g/l)	4.9 ± 0.9	4.8 ± 2.7	0.7221

To further validate the impact of NLR, we analyzed the results obtained from the Department of Obstetrics and Gynecology of Seoul National University Hospital admitted from 2016 to 2019. As shown in Table 6, the results showed that in patients with NLR <  = 3.7, systolic and diastolic blood pressure were increased, renal BUN and CREA values were increased. The coagulation functional parameters were different between NLR > 3.7 and NLR <  = 3.7 groups. The outcomes related with variables showed similar tendency in the SNUH validation set. These analyses showed that NLR < 3.7 may be a valuable predictor of organ dysfunctions in PE patients.

**Table 6:** Difference of index between the NLR > 3.7 and NLR ≤ 3.7 (Seoul National University Hospital, KOREA)

Index	> 3.7	$\le$ 3.7	*P* value
SBP (90–140 mmHg) (*n* = 146)	132 ± 27	140 ± 24	0.018(*)
DBP (60–90 mmHg) (*n* = 146)	82 ± 19	91 ± 17	0.001(*)
AST (1–40 IU/L) (*n* = 108)	38 ± 94	22 ± 10	0.378
ALT (1–40 IU/L) (*n* = 108)	28 ± 60	17 ± 13	0.798
TP (6.0–8.0 g/dL) (*n* = 105)	6.2 ± 0.6	6.1 ± 0.5	0.164
BUN (10–26 mg/dL) (*n* = 109)	9 ± 3	11 ± 5	0.013(*)
CREA (0.7–1.4 mg/dL) (*n* = 109)	0.55 ± 0.11	0.63 ± 0.18	0.004(*)
PT(10.6–12.9Sec) (*n* = 138)	10.2 ± 0.6	9.9 ± 0.6	0.006(*)
APTT(27.1–37.8Sec) (*n* = 138)	27.4 ± 2.4	28.4 ± 2.2	0.028(*)
FBGC(70–1120 mg/dl) (*n* = 103)	107 ± 28	96 ± 22	0.027(*)

## Discussion

The etiology and pathogenesis of PE have not been fully elucidated and the treatment of this disease is limited to lowering blood pressure or abrupt delivery. Since inflammation and hypertension are both critical to the development of PE, in this study, we retrospectively analyzed the correlations between hematological parameters such as NLR, MLR and PLR in PE patients with liver, kidney and coagulation functions. Furthermore, ROC analysis was performed to evaluate its diagnostic value and the causality/relationship. Results showed that Lym was increased in PE, as such, NLR, MLR and PLR were significantly reduced in PE patients, especially in sPE. NLR and PLR were negatively correlated with CREA and were positively correlated with TP and ALB. Only NLR was positively correlated with PT and APTT. Furthermore, NLR < 3.7 was the cut-off value for predicting the diagnosis of PE (blood pressure and kidney dysfunction) and organ functional indexes (liver and coagulation functions).

Over the last two decades, NLR from the peripheral blood is considered an important index to present systemic inflammation, as such, NLR is adversely related to clinical outcome in various diseases including cardiovascular diseases [28–30]. In PE, more and more studies show convincing evidence that NLR is related to PE patients [25, 31–34]. In general, the severity of PE is positively correlated with NLR, which implicates the roles of inflammatory status in the pathogenesis of this disease. Here, we have shown significant decreases of NLR in mPE and sPE patients. Our results also showed that among WBCs, Neu count was not altered in PE, but Lym count was significantly increased, result in lower NLR value in PE groups. In line with our study, a very recent study from Japanese group has suggested that lower NLR was associated with higher risk of preterm delivery of IPD in 76,835 pregnancies [35].

Similar to our results, Kim et al. and Canzoneri et al. also showed that Lym was increased in PE compared to normal pregnancy group [36, 37]. Lym exerts both pro-inflammatory and anti-inflammatory effects those are associated with the phenotypes in pregnancy. Anti-inflammatory T-regulatory cells (Tregs) are recognized to prevent the response of maternal immune system against fetal tissue and T-helper 17 cells (Th17) cells promote inflammation, autoimmunity and transplant rejection in humans. A significant increase in Th17 cells and/or decrease in Tregs has been reported in severe obstetric complications. Identification of Lym cells would be informative, but the reduction in the total Lym count in PE and the decrease in NLR suggest that such an inflammatory characteristic could play an important part in maintaining obstetric complications, such as PE [38]. Nevertheless, NLR is positively correlated with liver and coagulation parameters, especially in the sPE group, indicating that greater Neu could be linked to abnormal organ functions and high pro-inflammatory factors could detriment the pathological processes.

ROC analysis showed that the effective cut off value of NLR is 3.7. Notably, at the cut off value < 3.7, the clinical examination results (blood pressure, kidney as well as coagulation indexes) were indicative of PE patients in groups of China and Korea, confirm the diagnostic significance of NLR. Few studies have focused on reduced NLR, and there is no uniform conclusion on the use of NLR as a predictor of PE [25, 39–43]. Although difference in the sample sizes and ethnic origin of the patient groups may contribute to the discrepancies, our study claims the potential “J” shape impact of NLR in predicting PE. Taken together, the strength of the current study is to confirm that the increases in Lym in PE patients and the resultant lower NLR manifests inflammatory as well as organ functional changes.

Our research also showed that PLR and MLR were significantly lower in sPE than those in the normal pregnancy group. Similar to NLR, PLR was correlated with liver and kidney dysfunction in sPE, although PLR did not reach high enough sensitivity to be the diagnostic index for PE. Given that the activity of platelet and its changes during the progression of PE is essential in the disease progression, as implicated for PLR and mean platelet volume (MPV) in PE [17, 33], better understandings of platelet, PLR as well as MPV is important in understanding the interplays with NLR in organ damages and clinical outcome.

It is well known that leukocytes in peripheral blood were activated and it is associated with the hemodynamic changes during pregnancy and our results agree with the manifestations of these changes in PE patients. Activated immune cells adhere to vascular endothelial cells, increases capillary resistance by releasing toxic substances and damaging vascular endothelial cells. E.g. Shah et al. have shown that vascular tissue infiltrates a large number of Neu in PE patients [44]. In addition, Gupt et al. have shown that trophoblastic microparticles released from the placenta can effectively activate Neu and trigger the formation of Neu extracellular traps, further damaging vascular endothelial cells [45]. Reister et al. showed that Neu could be important bridges connecting zygotrophic cells and vascular endothelial cells, and induce systemic inflammatory responses in PE [46]. In addition, a large number of macrophages are shown to be infiltrated around the uterine spiral artery of PE patients, and trophoblastic infiltration is less than normal pregnant patients [47]. Macrophages can cause Th1 type cells to produce pro-inflammatory factors such as IL-2, IL-6, IL-8, TNF-α, which in turn, activate Neu and Lym to participate in inflammatory reactions and for trophoblastic cells to infiltrate the uterine spiral artery [48]. Combining prospective studies and the identification of immune cells and the released cytokine and chemokine profiles is warranted to bridging the gaps of upstream and downstream effectors for the pathological mechanisms.

## Conclusion

Our results reiterated the importance of NLR as an independent predictor of PE and associated organ dysfunction. The strength of the current study is that although NLR is reduced (due to Lym increment in PE group), the resultant lower NLR is valid in predicting PE diagnosis (blood pressure) and organ dysfunction (liver and coagulation impairment). Therefore, the study claims the caution for “J” shape of NLR in PE pathology. The limitation of the current study is not being able to further profiling and quantifying the immune cell types as in most retrospective studies. Large-scale studies (both retrospective and prospective) are required for better understandings of immune cell and platelet characteristics and their interplay for better protection and treatment of PE patients.

## Data Availability

The detailed datasets used and analyzed during the current study are available from the corresponding and first authors.

## Abbreviations

PE: Preeclampsia
NLR: Neutrophil to lymphocyte ratios
MLR: Monocyte to lymphocyte ratios
mPE: Mild PE
sPE: Severe PE
PLR: Platelet to lymphocyte ratio
TP: Total protein
ALB: Albumin
AST: Aspartate aminotransferase
ALT: Alanine aminotransferase
ALB: Albumin
TP: Total protein
TBIL: Total bilirubin
DBIL: Direct bilirubin
PT: Plasma prothrombin time
PTINR: Prothrombin time International normalized ratio
TT: Thrombin time
APTT: Activated partial thromboplastin time
FBGC: Fibrinogen quantification

## References

[1] Thornton C, Dahlen H, Korda A, Hennessy A (2013). The incidence of preeclampsia and eclampsia and associated maternal mortality in Australia from population-linked datasets: 2000–2008. Am J Obstet Gynecol.

[2] Shih T, Peneva D, Xu X, Sutton A, Triche E, Ehrenkranz RA, Paidas M, Steven W (2016). The Rising Burden of Preeclampsia in the United States Impacts Both Maternal and Child Health. Am J Perinatol.

[3] Kazemian E, Dorosty-Motlagh AR, Sotoudeh G, Eshraghian MR, Ansary S, Omidian M (2013). Nutritional status of women with gestational hypertension compared with normal pregnant women. Hypertens Pregnancy.

[4] Pecks U, Caspers R, Schiessl B, Bauerschlag D, Piroth D, Maass N, Rath W (2012). The Evaluation of the Oxidative State of Low-Density Lipoproteins in Intrauterine Growth Restriction and Preeclampsia. Hypertens Pregnancy.

[5] Milosevic-Stevanovic J, Krstic M, Radovic-Janosevic D, Stefanovic M, Antic V, Djordjevic I (2016). Preeclampsia with and without intrauterine growth restriction- two pathogenetically different entitles?. Hypertens Pregnancy.

[6] Dacaj R, Izetbegovic S, Stojkanovic G, Dreshaj S (2016). Elevated Liver Enzymes in Cases of Preeclampsia and Intrauterine Growth Restriction. Med Arch.

[7] Saito S, Sakai M, Sasaki Y, Tanebe K, Tsuda H, Michimata T (1999). Quantitative analysis of peripheral blood Th0, Th1, Th2 and the Th1:Th2 cell ratio during normal human pregnancy and preeclampsia. Clin Exp Immunol.

[8] Cornelius DC (2018). Preeclampsia: From Inflammation to Immunoregulation. Clin Med Insights Blood Disord..

[9] Faas MM, Spaans F, Vos PD (2014). Monocytes and Macrophages in Pregnancy and Pre-Eclampsia. Front Immunol.

[10] Faas MM, Vos P (2017). Maternal Monocytes in Pregnancy and Preeclampsia in Humans and in Rats. J Reprod Immunol.

[11] Elgari MM, Khabour OF, Alhag SM (2019). Correlations Between Changes in Hematological Indices of Mothers With Preeclampsia and Umbilical Cord Blood of Newborns. Clin Exp Hypertens.

[12] Conrad KP, Benyo DF (1997). Placental Cytokines and the Pathogenesis of Preeclampsia. AJRI.

[13] Lam C, Lim KH, Karumanchi SA (2005). Circulating Angiogenic Factors in the Pathogenesis and Prediction of Preeclampsia. Hypertension.

[14] Morton JS, Cooke CL, Davidge ST (2016). In Utero Origins of Hypertension: Mechanisms and Targets for Therapy. Physiol Rev.

[15] Bhutta H, Agha R, Wong J, Tang TY, Wilson YG, Walsh SR (2011). Neutrophil-lymphocyte Ratio Predicts Medium-Term Survival Following Elective Major Vascular Surgery: A Cross-Sectional Study. Vasc Endovascular Surg.

[16] Hwang SY, Shin TG, Jo IJ, Jeon K, Suh GY, Lee TR, Yoon H, Cha WC, Sim MS (2017). Neutrophil-to-lymphocyte Ratio as a Prognostic Marker in Critically-Ill Septic Patients. Am J of Emerg Med.

[17] Seng JJB, Kwan YH, Low LL, Thumboo J, Fong WSW (2018). Role of Neutrophil to Lymphocyte ratio (NLR), Platelet to Lymphocyte ratio (PLR) and Mean Platelet Volume (MPV) in Assessing Disease Control in Asian Patients With Axial Spondyloarthritis. Biomarkers.

[18] Hu Y, Li H, Yan R, Wang C, Wan Y, Zhang C, Liu M, Zhou T, Zhu W, Zhang H, Dong N, Wu Q (2018). Increased Neutrophil Activation and Plasma DNA Levels in Patients with Pre-Eclampsia. Thromb haemost.

[19] Ji F, Liang Y, Fu SJ, Guo ZY, Shu M, Shen SL, Li SQ, Peng BG, Liang LJ, Hua YP (2016). A Novel and Accurate Predicitor of Survival for Patients with Hepatocellular Carcinoma After Surgical Resection: The Neutrophil to Lymphocyte Ratio (NLR) Combined with the Aspartate Aminotransferase / Platelet Count Ratio Index (APRI). BMC Cancer.

[20] Sisti G, Williams B (2019). Body of Evidence in Favor of Adopting 130/80mmHg as New Blood Pressure Cut-off for All the Hypertensive Disorders of Pregnancy. Medicina (kaunas).

[21] Oylumlu M, Ozler A, Yildiz A, Oylumlu M, Acet H, Polat N, Soydinc HE, Yuksel M, Ertas F (2014). New Inflammatory Markers in Pre-Eclampsia: Echocardiographic Epicardial Fat Thickness and Neutrophil to Lymphocyte Ratio. Clin Exp Hypertens..

[22] Gogoi P, Sinha P, Gupta B, Firmal P, Rajaram S (2019). Neutrophil-to-lymphocyte Ratio and Platelet Indices in Pre-Eclampsia. Int J Gynecol Obstet.

[23] Serin S, Avci F, Ercan O, Bakacak M, Kiram H, KÖstü B (2016). Is Neutrophil Lymphocyte Ratio a Useful Marker to Predict the Severity of Pre-Eclampsia?. Pregnancy Hypertens..

[24] Wang J, Zhu QW, Cheng XY, Liu JY, Zhang LL, Tao YM, Cui YB, Wei Y (2019). Assessment Efficacy of Neutrophil-Lymphocyte Ratio and Monocyte-Lymphocyte Ratio in Preeclampsia. J Reprod Immunol.

[25] Yücel B, Ustun B (2017). Neutrophil to Lymphocyte Ratio, Platelet to Lymphocyte Ratio, Mean Platelet Volume, Red Cell Distribution Width and Plateletcrit in Preeclampsia. Pregnancy Hypertens.

[26] Cintesun E, Cintesun FNI, Ezveci H, Akyürek F, Celik C (2018). Systemic inflammatory response markers in preeclampsia. J Lab Physicians.

[27] Okoye HC, Madu AJ, Korubo K, Efobi C, Eze OE, Obodo O, Okereke K, Ilechukwu G (2019). Correlates of neutrophil/lymphocyte, platelet/lymphocyte, and platelet/neutrophil ratios of neonates of women with hypertensive disease of pregnancy with neonatal birth outcomes. Hypertens Pregnancy.

[28] Uthamalingam S, Patvardhan EA, Subramanian S (2011). Utility of the neutrophil to lymphocyte ratio in predicting long-term outcomes in acute decompensated heart failure. Am J Cardiol.

[29] Tonyali S, Ceylan C, Yahsi S, Karakan MS (2018). Does neutrophil to lymphocyte ratio demonstrate deterioration in renal function?. Ren Fail.

[30] Norlander AE, Madhur MS, Harrison DG (2018). The immunology of hypertension. J Exp Med.

[31] Lurie S, Frenkel E, Tuvbin Y (1998). Comparison of the differential distribution of leukocytes in preeclampsia versus uncomplicated pregnancy. Gynecol Obstet Invest.

[32] Sisti G, Faraci A, Silva J, Upadhyay R (2019). Neutrophil-to-lymphocyte ratio, platelet-to-lymphocyte ratio, and routine complete blood count components in HELLP syndrome: a matched case control study. Medicina.

[33] Mannaerts D, Heyvaert S, De Cordt C, Macken C, Loos C, Jacquemyn Y (2019). Are neutrophil/lymphocyte ratio (NLR), platelet/lymphocyte ratio (PLR), and/or mean platelet volume (MPV) clinically useful as predictive parameters for preeclampsia?. J Matern Fetal Neonatal Med.

[34] Gogoi P, Sinha P, Gupta B, Firmal P, Rajaram S (2019). Neutrophil-to-lymphocyte ratio and platelet indices in pre-eclampsia. Int J Gynaecol Obstet.

[35] Morisaki N, Piedvache A, Nagata C, Michikawa T, Morokuma S, Kato K, Sanefuji M, Shibata E, Tsuji M, Shimono M, Ohga S, Kusuhara K, Japan Environment and Children’s Study Group (2021). Maternal blood count parameters of chronic inflammation by gestational age and their associations with risk of preterm delivery in the Japan Environment and Children's Study. Sci Rep..

[36] Kim MA, Han GH, Kwon JY, Kim YH (2018). Clinical Significance of Platelet-To-Lymphocyte Ratio in Women with Preeclampsia. J Reprod Immunol.

[37] Canozoneri B, Lewis DF, Groome L, Wang Y (2009). Increased Neutrophil Numbers Account for Leukocytosis in Women with Preeclampsia. Am J perinatol.

[38] El Shahaway AA, Elhady RRA, Abdelrhman AA, Yahia S (2019). Role of maternal serum interleukin 17 in preeclampsia: diagnosis and prognosis. J Inflamm Res.

[39] Kirbas A, Ersoy AO, Daglar K, Dikici T, Biberoglu EH, Kirbas O, Danisman N (2015). Prediction of preeclampsia by first trimester combined test and simple complete blood count parameters. J Clin Diagn Res..

[40] Yavuzcan A, Caglar M, Ustün Y, Dilbaz S, Ozdemir I, Yildiz E, Ozbilgec S, Kumru S (2014). Mean platelet volume, neutrophil-lymphocyte ratio and platelet-lymphocyte ratio in severe preeclampsia. Ginekol Pol.

[41] Kurtoglu E, Kokcu A, Celik H, Tosun M, Malatyalioglu E (2015). May ratio of neutrophil to lymphocyte be useful in predicting the risk of developing preeclampsia? A pilot study. J Matern Fetal Neonatal Med..

[42] Gerzer C, Ekin A, Ertas IE, Ozeren M, Solmaz U, Mat E, Taner CE (2016). High first-trimester neutrophil-to-lymphocyte ratio and platet-to-lymphocyte ratios are indicators for early diagnosis of preeclampsia. Ginekol Pol.

[43] Cakmak HA, Dincgez Cakmak B, Abide Yayla C, Coskun E, Erturk M, Keles I (2017). Assessment of relationship between novel inflammatory makers and presence and severity of preeclampsia:epicardial fat thickness, pentraxin-3, and neutrophil-to-lymphocyte ratio. Hypertens Pregnancy.

[44] Shah TJ, Walsh SW (2007). Activation of NF-kB and Expression of COX-2 in Association with Neutrophil/Infiltration in Systemic Vascular Tissue of Women with Preeclampsia. Am J Obstet Gynecol.

[45] Gupta AK, Hasler P, Holzgreve W, Gebhardt S, Hahn S (2005). Induction of Neutrophil Extracellular DNA Lattices by Placental Microparticles and IL-8 and Their Presence in Preeclampsia. Hum Immunol.

[46] Reister F, Frank HG, Kingdom JC, Heyl W, Kaufmann P, Rath W, Huppertz B (2001). Macrophage- induced Apoptosis Limits Endovascular Trophoblast Invasion in the Uterine Wall of Preeclamptic Women. Lab Invest.

[47] Sunbul M, Gerin F, Durmus E, Kivrak T, Sari I, Tigen K, Cincin A (2014). Neutrophil to Lymphocyte and Platelet to Lymphocyte Ratio in Patients with Dipper Versus Non-Dipper Hypertension. Clin Exp Hypertens.

[48] Bogdan C (2011). Regulation of Lymphocytes by Nitric Oxide. Suppression Regul Immune Responses.

